# A poisoned apple: First insights into community assembly and networks of the fungal pathobiome of healthy-looking senescing leaves of temperate trees in mixed forest ecosystem

**DOI:** 10.3389/fpls.2022.968218

**Published:** 2022-11-03

**Authors:** Benjawan Tanunchai, Li Ji, Simon Andreas Schroeter, Sara Fareed Mohamed Wahdan, Panadda Larpkern, Ann-Sophie Lehnert, Eliane Gomes Alves, Gerd Gleixner, Ernst-Detlef Schulze, Matthias Noll, François Buscot, Witoon Purahong

**Affiliations:** ^1^ UFZ-Helmholtz Centre for Environmental Research, Department of Soil Ecology, Halle (Saale), Germany; ^2^ Bayreuth Center of Ecology and Environmental Research (BayCEER), University of Bayreuth, Bayreuth, Germany; ^3^ School of Forestry, Central South of Forestry and Technology, Changsha, China; ^4^ Max Planck Institute for Biogeochemistry, Biogeochemical Processes Department, Jena, Germany; ^5^ Department of Botany and Microbiology, Faculty of Science, Suez Canal University, Ismailia, Egypt; ^6^ Bodhivijjalaya College, Srinakharinwirot University, Nakhon Nayok, Thailand; ^7^ Institute for Bioanalysis, Coburg University of Applied Sciences and Arts, Coburg, Germany; ^8^ German Centre for Integrative Biodiversity Research (iDiv), Halle-Jena-Leipzig, Leipzig, Germany

**Keywords:** foliar fungal pathogens, next generation sequencing, deterministic processes, stochastic processes, homogenising dispersal, ecological drift

## Abstract

Despite the abundance of observations of foliar pathogens, our knowledge is severely lacking regarding how the potential fungal pathobiome is structured and which processes determine community assembly. In this study, we addressed these questions by analysing the potential fungal pathobiome associated with the senescing leaves and needles of 12 temperate tree species. We compared fungal plant pathogen load in the senescing leaves/needles and demonstrated that healthy-looking leaves/needles are inhabited by diverse and distinct fungal plant pathogens. We detected 400 fungal plant pathogenic ASVs belonging to 130 genera. The fungal plant pathogenic generalist, *Mycosphaerella*, was found to be the potential most significant contributor to foliar disease in seedlings. The analyses of assembly process and co-occurrence network showed that the fungal plant pathogenic communities in different tree types are mainly determined by stochastic processes. However, the homogenising dispersal highly contributes in broadleaf trees, whereas ecological drift plays an important role in coniferious trees. The deterministic assembly processes (dominated by variable selection) contributed more in broadleaf trees as compared to coniferous trees. We found that pH and P level significantly corresponded with fungal plant pathogenic community compositions in both tree types. Our study provides the first insight and mechanistic understanding into the community assembly, networks, and complete taxonomy of the foliar fungal pathobiome in senescing leaves and needles.

## Introduction

Mixed forest ecosystems have recently received considerable attention due to their advantages over monospecific forests in the context of global climate change but also in relation with both economic and ecological aspects (Clasen et al., 2011; Gamfeldt et al., 2013; Almeida et al., 2018). Mixed forests have been reported to have a better risk–return relation compared to monocultures, which are associated with higher risks and lower returns (Clasen et al., 2011). The net present value (defined as appropriately discounted and summed net revenues gained or caused by the management) of mixed beech forests can reach the annual gains up to 113 € ha^−1^ yr^−1^ as compared with 72 € ha^−1^ yr^−1^ for beech monocultures (Clasen et al., 2011). In addition to this economic perspective, mixed forests, especially those based on natural regeneration (forests that are allowed to maintain natural growth cycles with minimal human intervention), exhibit ecological advantages over monospecific forests, including promoting biodiversity and ecosystem functions, maintaining tree genetic diversity, increasing resilience to climate change, and enhancing high resistance to biotic and abiotic hazards (Griess et al., 2012; Almeida et al., 2018; Ehbrecht et al., 2021). To date, mixed forest ecosystem covers more than 200 million ha worldwide or about 5% of the global forest area (FAO and UNEP, 2020). In Germany, the mixed forest area increased to ~58% of the total forest area in 2012 (Wilke, 2017).

In mixed forest ecosystems, niche partitioning and competitions (intra- and interspecific competitions) play important roles in shaping tree species community composition and diversity. The sources for fungal leaf pathogens are shedding leaves and/or fungal bodies of pathogenic fungi (Sánchez Márquez et al., 2011; Bayandala et al., 2016). Senescing leaves may attract diverse fungal functional groups, including fungal plant pathogens (Tanunchai et al., 2022). However, such knowledge in temperate tree species, including their taxonomy, assembly processes, specificity and factors corresponding to their community composition, is still largely unexplored. For evergreen coniferous trees, senescing needles shed over years and thus represent continuous input of fungal pathogens to the soil system. Conversely, senescing needles and leaves of coniferous and broadleaves deciduous trees mainly shed during autumn, creating a large seasonal wave of fungal pathogens into the soil system. Interactions among microbial taxa can be complex as different taxa can express antagonistic, competitive, or mutualistic interactions (Montoya et al., 2006; Deng et al., 2012; Tyc et al., 2014). Investigating interactions among different microbial taxa within a community and their responses to environmental changes enables a better understanding of ecological mechanisms and outcomes (Sheng et al., 2019). To achieve this comprehensive analytic perspective, ecological network approaches have been intensively applied to investigate the complexity of interactions among different microbial taxa (Montoya et al., 2006; Deng et al., 2012; Toju et al., 2014). In general, environmental filtering by means of substrate physicochemical properties is considered as a main process shaping the community in plant debris (Purahong et al., 2016); however, the community assembly may be largely explained by stochastic processes (Abrego, 2021). This issue has never been addressed for the senescing leaves of diverse temperate tree species.

Despite the importance of foliar fungal pathogens in regulating tree diversity and community composition in temperate forests, our knowledge on their ecology and community assembly remains limited. Specifically, it remains unclear which factors shape foliar fungal pathobiome community composition, which processes (stochastic vs. deterministic) determine their community assembly, and how their community structure is organized. In this study, we used a high–resolution molecular approach (Next Generation Sequencing) to obtain a better understanding of the fungal pathobiome of healthy-looking senescing leaves and needles among 12 temperate tree species growing in a managed mixed forest at the Central Germany. We aimed to determine (i) which ecological processes determine the pathobiome community assembly, (ii) how foliar fungal pathobiome communities are structured, (iii) which factors determine community composition of the foliar fungal pathogens, and (iv) which tree species that act as fungal plant pathogen hub in this forest ecosystem.

## Materials and methods

### Study site and sampling

The study site is located in a managed mixed forest of Thuringia, Germany (51°12’N 10°18’E) and is characterized by mean annual precipitation from 600 to 800 mm, mean annual temperature from 6 to 7.5°C, and elevations from 100 to 494 m above sea level. The main soil type is Cambisol on limestone as bed-rock. The soil pH is weakly acidic (5.1 ± 1.1; mean ± SD). In October 2019, a minimum of 200-g healthy-looking senescing leaves and needles of 12 mature tree species (5 true replicates/tree individual per tree species, in total 60 samples) were collected in a separate sterile plastic bag with new clean gloves. In this current study, we characterized the healthy-looking senescing leaf as green leaves without visible leaf disease symptoms from branches at the lower part of the crown of the mature tree (3 years old). These 12 tree species include 8 deciduous broadleaf (including *Acer pseudoplatanus, Carpinus betulus, Fagus sylvatica, Fraxinus excelsior, Populus hybrid, Prunus avium, Quercus robur, and Tilia cordata*), 3 evergreen (including *Picea abies, Pinus sylvestris, and Pseudotsuga menziesii*), and 1 deciduous (*Larix decidua*) coniferous tree species. Leaf samples were transported on ice to the laboratory within 3 h and stored at −80°C for further analysis.

### DNA extraction and illumina sequencing

To prepare for deoxyribonucleic acid (DNA) extraction, up to 10 healthy-looking leaves and needles from 5 branches per individual tree were subsampled. Leaf and needle samples were washed three times with 0.1% sterile Tween to remove loosely attached dust particles. The samples were then washed three to five times using deionized water and incubated for 1 h in sterile water to remove the Tween bubbles. By washing leaves with Tween solution, the endophytic and strongly attached epiphytic microorganisms were subjected to the DNA extraction. Leaf and needle samples were ground using liquid nitrogen and sterile nails, homogenized, and stored at −20°C for further analysis. The DNA extraction of senescing leaves and needles and associated microbes was performed using DNeasy PowerSoil Kit (Qiagen, Hilden, Germany) and a Precellys 24 tissue homogenizer (Bertin Instruments, Montigny-le-Bretonneux, France) according to the manufacturer’s instructions. The presence and quantity of genomic DNA was checked using NanoDrop ND-1000 spectrophotometer (Thermo Fisher Scientific, Dreieich, Germany), and the extracts were stored at −20°C.

Leaf-associated fungi were characterized by fungal internal transcribed spacer (ITS)-based amplicon sequencing on the Illumina MiSeq sequencing platform, as outlined previously (Weißbecker et al., 2020; Tanunchai et al., 2022). To establish fungal amplicon libraries, the fungal ITS2 gene was amplified using the fungal primer pair fITS7 [5′-GTGARTCATCGAATCTTTG-3′] (Ihrmark et al., 2012) and ITS4 [5′-TCCTCCGCTTATTGATATGC-3′] (White et al., 1990) with Illumina adapter sequences. Amplifications were performed using 20-µL reaction volumes with 5× HOT FIRE Pol Blend Master Mix (Solis BioDyne, Tartu, Estonia). The amplified products were visualized by gel electrophoresis and purified using an Agencourt AMPure XP kit (Beckman Coulter, Krefeld, Germany). Illumina Nextera XT Indices were added to both ends of the fungal amplicons. The products from three technical replicates were then pooled in equimolar concentrations. Paired-end sequencing (2 × 300 base pair) was performed on the pooled polymerase chain reaction products using a MiSeq Reagent kit v3 on an Illumina MiSeq system (Illumina Inc., San Diego, CA, United States) at the Department of Soil Ecology, Helmholtz Centre for Environmental Research, Germany. The ITS ribosomal ribonucleic acid (rRNA) gene sequences are deposited in the National Center for Biotechnology Information (NCBI) Sequence Read Archive under the accession number PRJNA753096.

### Bioinformatics

The ITS ribosomal DNA (rDNA) sequences corresponding to the forward and reverse primers were trimmed from the demultiplexed raw reads using cutadapt (Martin, 2011). Paired-end sequences were quality-trimmed, filtered for chimeras, and merged using the DADA2 package (Callahan et al., 2016) through the dadasnake pipeline (Weißbecker et al., 2020). Assembled reads fulfilling the following criteria were retained for further analysis: a minimum length of 70 nucleotides (nt), quality scores at least equal to 9 with maximum expected error score of 5 for forward and reverse sequences and no ambiguous nucleotides. Merging was conducted with an allowance for 2 mismatches and a minimum overlap of 20 nt required for fungal sequences. High-quality reads of fungi were clustered into 2480 amplicon sequence variants (ASVs) after chimera removal. Fungal ASVs were classified against the UNITE v7.2 database (Kõljalg et al., 2013). ASV sets were classified using the Bayesian classifier (Wang et al., 2007) in the mothur classify.seqs command with a cut-off of 60. Rare ASVs (singletons), which may represent artificial sequences, were removed. 2,451 fungal ASVs with minimum sequencing depths of 21,967 sequences per sample were obtained. We used a Mantel test based on Bray–Curtis distance measure with 999 permutations to assess the correlation between the whole matrix and a rarified matrix for fungal data sets. The results indicated that the rarefaction dataset highly represents the whole fungal matrix (*R*_Mantel_ = 0.997, *P* = 0.001). Among these, 400 ASVs were classified as potential plant pathogens according to primary lifestyle in FungalTraits database (Põlme et al., 2020). The information on fungal plant pathogenic ASVs with average relative abundances (ranged from 8–67%) and taxonomic information is provided in **Supplementary Table S1**. The 400 fungal plant pathogenic ASVs were again rarified to the minimum read of 1,245, except one and two replicates of *P. avium* and *P. abies*, were not rarified, to confirm the consistency of the fungal plant pathogenic ASV richness.

### Investigation of the fate of fungal plant pathogens

We preliminary investigated the fate of plant pathogens in senescing leaves and needles after 200 and 400 days of the decomposition. The collected senescing leaves and needles were oven dried at 25°C for 14 days. Three grams of oven-dried senescing leaves and needles were filled into a nylon bag (2 mm mesh, 5 mm holes), placed back under the same tree individuals (mother tree) and allowed to decompose. After 200 and 400 days of decomposition, leaf/needle samples were collected in a separate sterile plastic bag with new clean gloves and transported on ice to the laboratory within 3 h and stored at −80°C for further analysis. The decomposing leaf/needle samples were processed with the same procedures for sample preparation, DNA extraction as well as bioinformatics as described above.

### Leaf physiochemical analyses

To obtain water-leachable components, senescing leaf and needle samples were shaken in 30 mL milliQ water for 1 h in falcon tubes, centrifuged for 5 min at 3500 rpm, decanted, and filtered. The remaining leaf/needle material was dried for two weeks at 40°C to determine dry weight, which was used as reference for all subsequent qualifications. Leachate pH was determined using pH paper with a scale precision of 0.2 units. Organic nitrogen (N_org_) was calculated as the difference: N_org_ = Total nitrogen (TN) – mineralized nitrogen (N_Min_). TN was analyzed using a sum parameter analyzer with high temperature combustion and chemiluminescence detection (Mitsubishi TN-100; a1 envirosciences, Düsseldorf, Germany). For N_Min_ quantification, a flow injection analyzer (Quikchem QC85S5; Lachat Instruments, Hach Company, Loveland CO, USA) was used with corresponding manifolds to measure ammonium nitrogen ${\text{N}}_{{\text{NH}}_{4}^{+}}$ , nitrite nitrogen ${\text{N}}_{{\text{NO}}_{2}^{-}}$ , and nitrate- plus nitrite nitrogen ${\text{N}}_{{\text{NO}}_{3}^{-}+{\text{NO}}_{2}^{-}}$ content. Dissolved organic carbon (DOC) was quantified as non-purgeable organic carbon with a sum parameter analyzer using high-temperature combustion and infrared detection (vario TOC cube, Elementar Analysensysteme GmbH, Langenselbold, Germany). The nutrient content, Ca, Fe, K, Mg, and P analyses were carried out using inductively coupled plasma–optical emission spectrometry “Arcos” (Spectro, Kleve, Germany) equipped with a 27.12 MHz free-running LDMOS generator and ORCA optical system. The complete methods for leaf physiochemical analyses are provided in the **Supplementary Material**.

### Network analyses and generalist/ specialist determination

The fungal plant pathogenic network analysis was constructed based on Random Matrix Theory using the molecular ecological network analysis pipeline (http://ieg4.rccc.ou.edu/mena/). Spearman rank correlation coefficients was analyzed between any two pairs of ASVs based on sequencing reads. The intra-module connectivity value (*Zi*) and inter-module connectivity value (*Pi*) for each node were used to identify keystone species in the network. For detailed information regarding theories, algorithms, and procedures, refer to Deng et al., 2012 and Zhou et al., 2011. The fungal plant pathogenic networks were visualized using Gephi v0.9.2. The co-occurrence network between tree species and fungal plant pathogens was visualized in Cytoscape v. 3.8.0. Specialist/generalist classification of the taxonomic dataset in this study was performed using EcolUtils package in R version 4.0.4 based on niche width and permutation algorithms. R code for specialist/generalist classification in this study is provided in **Supplementary Material**.

### Community assembly analyses

To quantify the relative proportion of deterministic and stochastic processes in community assembly, the phylogenetic normalized stochasticity ratio (pNST) and beta nearest taxon indices (βNTI) based on the null model theory were calculated using ‘iCAMP’ package in R (Stegen et al., 2013; Ning et al., 2020; Sun et al., 2021). The fungal ITS gene sequences obtained by Illumina sequencing have been recently used to construct the phylogenetic and null model analyses for determining the assembly processes of fungal communities (Gyeong et al., 2021; Wang et al., 2022; Zhao et al., 2022). Although 18S nuclear ribosomal small subunit rRNA gene (SSU) is more appropriate and commonly applied to construct the phylogenetic tree of fungi than the ITS gene, it has lower hypervariable domains in fungi (Schoch et al., 2012). Thus, it had inferior taxonomic resolution as compared with the ITS. In this current study, we focus more on the taxonomic identification of fungi, so the ITS region was chosen as the region for detecting plant pathogenic fungi. Nevertheless, the ITS regions (full ITS, ITS1, and ITS2) have been successfully used to construct relatively reliable phylogenetic tree of many fungal genera (Porter and Brian Golding, 2011; Dissanayake et al., 2018; Purahong et al., 2019). Thus, the results from pNST derived from the ITS sequences should be interpreted with caution. In addition to βNTI, the Raup-Crick (RC_bray_) null model based on Bray-Curtis dissimilarity was further calculated to quantify dispersal-based stochastic ecological processes generating the turnover of community composition (Stegen et al., 2015). Briefly, for pNST index, deterministic and stochastic assembly were determined when pNST< 50% and pNST > 50%, respectively. For the βNTI, homogeneous and variable selection are indicated by βNTI< −2 and βNTI > +2, respectively. The relative importance of dispersal limitation and homogenizing dispersal processes were assessed by |βNTI|< 2 but RCbray > +0.95 and RCbray< −0.95, respectively, and the undominated process was estimated by |βNTI|< 2 and |RCbray|< 0.95 (Stegen et al., 2015). Apart from pNST analysis, we have analyzed microbial assembly based on the taxonomic normalized stochasticity ratio (tNST) according to Ning et al., 2019.

### Statistical analyses

The datasets were tested for normality using the Jarque–Bera test and for equality of group variances using *F*-test (for two datasets) and Levene’s test (for more than two datasets). The differences among the generalists, and specialists were tested using Kruskal-Wallis test and one-way analysis of variance (ANOVA) for the data sets with non- and equality of variance, respectively. The statistical differences between the generalists and specialist in each tree species were performed using *t*-test. The statistical differences of ASV richness among different tree species were performed using one-way ANOVA with Tukey’s *post-hoc* test. Effects of tree species on fungal plant pathogenic community composition were tested using non-metric multidimensional scaling (NMDS), permutational multivariate ANOVA, and analysis of similarities based on observed relative abundance and the Bray–Curtis distance measure as well as presence/absence data and the Jaccard distance measure, over 999 permutations. The relationship between fungal plant pathogenic community compositions and different factors was analyzed using a goodness-of-fit statistic based on observed relative abundance and the Bray–Curtis distance measure as well as presence/absence data and the Jaccard distance measure. To differentiate the effect of an individual factor as well as their combining effect, we performed the variance partitioning analysis. The amounts of variation in fungal plant pathogenic community compositions, explained by various factors, were estimated through variation partitioning using the Vegan package in R. R code for goodness-of-fit statistic and variance partitioning analysis in this study is provided in **Supplementary Material**. All statistical analyses were performed using PAST version 2.17 (Hammer et al., 2001), R, and RStudio version 4.0.4 (RStudio Team, 2019).

## Results

### Fungal pathobiome characteristics

**Figure 1:**
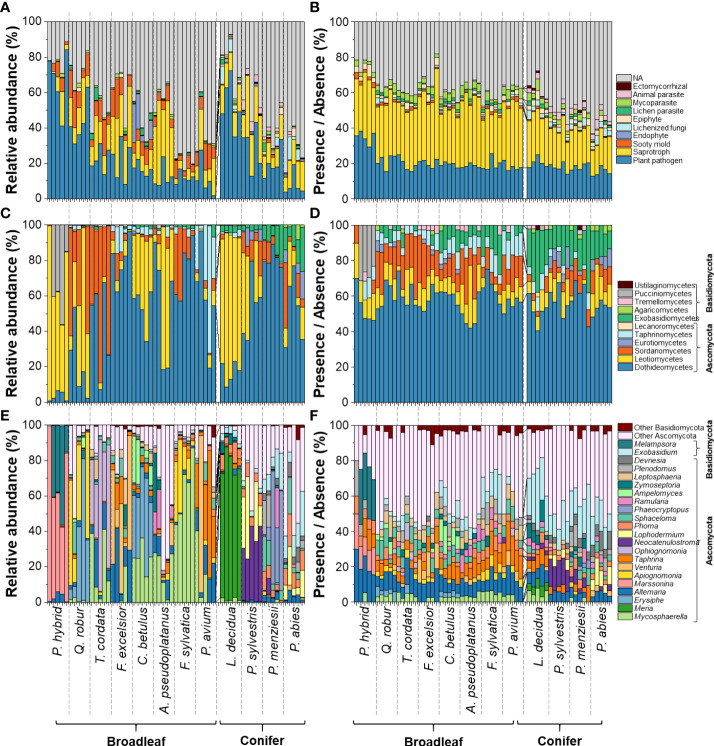
Compositions of fungal functional groups **(A, B)** and fungal plant pathogens **(C–F)** associated with senescing leaves of 12 temperate tree species at class **(C, D)** and genus levels **(E,F)**, considering only classes with ≥ 8 ASVs or relative abundances ≥ 2%. Remaining fungal classes and genera were pooled as “others”. Analysis based on relative abundance (left panel) and presence/absence data (right panel).

In this study, the fungal community compositions associated with healthy-looking leaves and needles of 12 temperate tree species were analysed (**Figure 1**). We detected 400 fungal plant pathogenic ASVs belonging to 130 genera (**Supplementary Table S1**). Fungal plant pathogens contributed the highest proportion among all fungal functional groups, especially in leaves and needles of *P. hybrid*, *L. decidua*, and *Q. robur* with Leotiomycetes (represented by *Marssonina*, *Phoma*, *Meria*, and *Erysiphe*) contributing up to 99, 87, and 81% of fungal plant pathogens, respectively (**Figure 1**). Leaves and needles of other tree species were dominated by fungal plant pathogenic genera in Dothideomycetes, including *Mycosphaerella*, *Phoma*, *Alternaria*, and *Venturia*. Among these 400 fungal plant pathogenic ASVs, 214 ASVs and 16 ASVs revealed saprotrophic and endophytic lifestyle as secondary lifestyles, respectively. 75 ASVs have the foliar endophytic interaction capability (**Supplementary Table S1**).

### Diverse fungal plant pathogens detected in 12 temperate tree species

**Figure 2:**
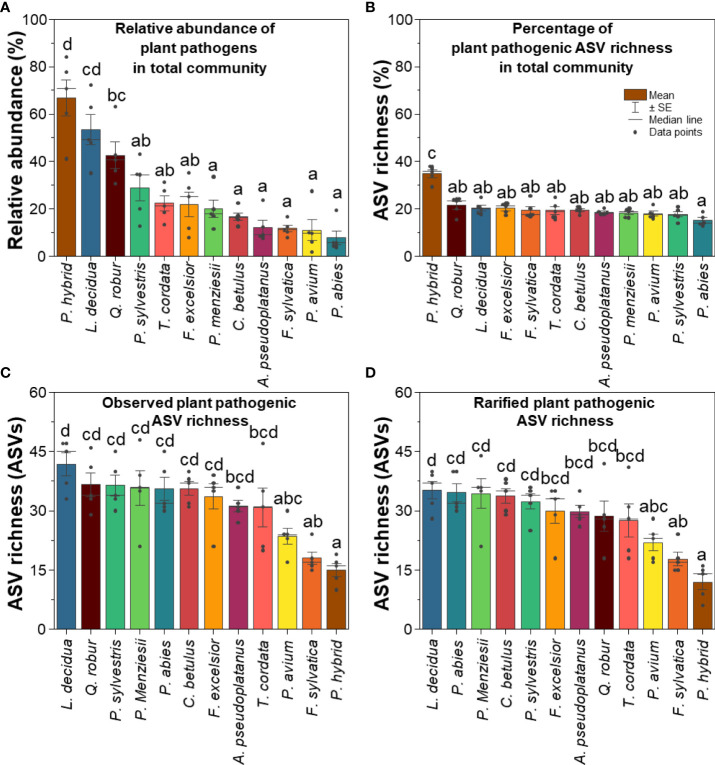
**(A)** Relative abundance, **(B)** percentage of ASV richness of fungal plant pathogens in total fungal community, **(C)** observed, and **(D)** rarified fungal plant pathogenic ASV richness. Different lowercase letters indicate significant differences according to one-way ANOVA incorporating Tukey’s *post-hoc* test.

In line with the relative abundance data, *P. hybrid* also showed the highest percentage of fungal plant pathogenic ASV richness relative to the total fungal groups, and *P. abies* revealed the lowest percentage of ASV richness (**Figure 2**). *Larix decidua* harbored a high relative abundance and the highest fungal plant pathogenic ASV richness (Figsure 2). In contrast to *L. decidua*, *P. hybrid* revealed the highest relative abundance but the lowest ASV richness of fungal plant pathogens.

### Community compositions and specificity of fungal plant pathogens

**Figure 3:**
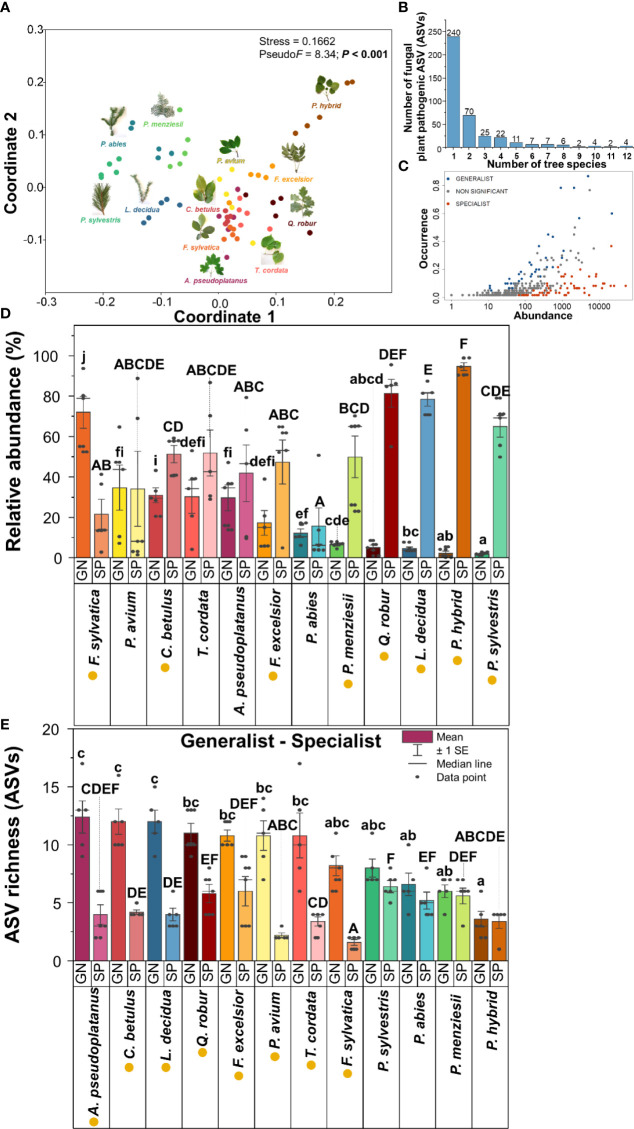
Fungal plant pathogenic community compositions and their specificity: NMDS ordinations of fungal plant pathogenic community compositions based on relative abundance and Bray–Curtis distance similarity **(A)**, number of fungal plant pathogenic ASVs detected in various number of tree species **(B)**, the abundance and the occurrence of generalists and specialists of fungal plant pathogens **(C)**, relative abundance **(D)**, and ASV richness **(E)** of fungal plant pathogenic generalists and specialists in each tree species. Generalist and specialist classification refers only to our taxonomic data from this local scale experiment. Capital and lowercase letters in panels **(D, E)** indicate statistically significant differences (Kruskal–Wallis test and ANOVA were performed for the data sets with non- and equality of variance, respectively) among fungal plant pathogenic specialists (SP) and generalists (GN), respectively. The yellow circle in the front of tree species name indicates statistically significant differences (*t*-test) between fungal plant pathogenic generalist and specialist in each tree species.

Leaves and needles of 12 temperate tree species harboured distinct fungal plant pathogenic community compositions based on both relative abundance and presence/absence data (**Figure 3**, **Supplementary Figure S1** and **Supplementary Tables S2A, B**). In this study, 240 ASVs (accounting for 60% of the total fungal plant pathogenic ASVs) were specific to one tree species. Only four fungal plant pathogenic ASVs (*Alternaria* ASV55, ASV56, ASV86, and *Neoascochyta* ASV227) were detected across all 12 temperate tree species (**Figure 3B** and **Supplementary Table S1**). In this current study, fungal plant pathogenic generalists and specialists showed significantly different relative abundances and richness across most studied temperate tree species (**Figures 3D, E**). The highest relative abundance of fungal plant pathogenic generalists and specialists was found in *F. sylvatica* and *P. hybrid*, respectively (**Figure 3D**). *A. pseudoplatanus* exhibited the highest ASV richness of fungal plant pathogenic generalists, followed by *C. betulus* and *L. decidua* (**Figure 3E**). Remarkably, *L. decidua* and *Q. robur* harboured significantly higher ASV richness of generalists as compared with specialists, and *P. hybrid* also harboured similar ASV richness of generalists and specialists, but 78–95% relative abundance of their fungal plant pathogens belonged to specialists.

### Are there any tree species that act as fungal plant pathogen hub in this forest ecosystem?

In general, no specific mature tree species were found to behave as a fungal plant pathogen hub. However, some tree species harboured significantly more fungal plant pathogens. *P. menziesii* harboured the highest number of fungal plant pathogenic ASVs (97 ASVs), followed by *Q. robur* (96 ASVs), *L. decidua* (95 ASVs), *A. pseudoplatanus* (81 ASVs), and *F. excelsior* (80 ASVs). *P. hybrid* harboured the lowest number of fungal plant pathogenic ASVs (29 ASVs). Among 400 fungal plant pathogenic ASVs, 240 ASVs connected with only one tree species (**Supplementary Figure S2**). 156 fungal plant pathogenic ASVs build network with two to 11 tree species and four ASVs were detected in all 12 tree species. Furthermore, some tree species harboured significantly more fungal plant pathogenic generalists, including *A. pseudoplatanus* (25 ASVs), *F. excelsior* (23 ASVs), and *Q. robur* (22 ASVs).

### Assembly processes: Deterministic vs. stochastic

**Figure 4:**
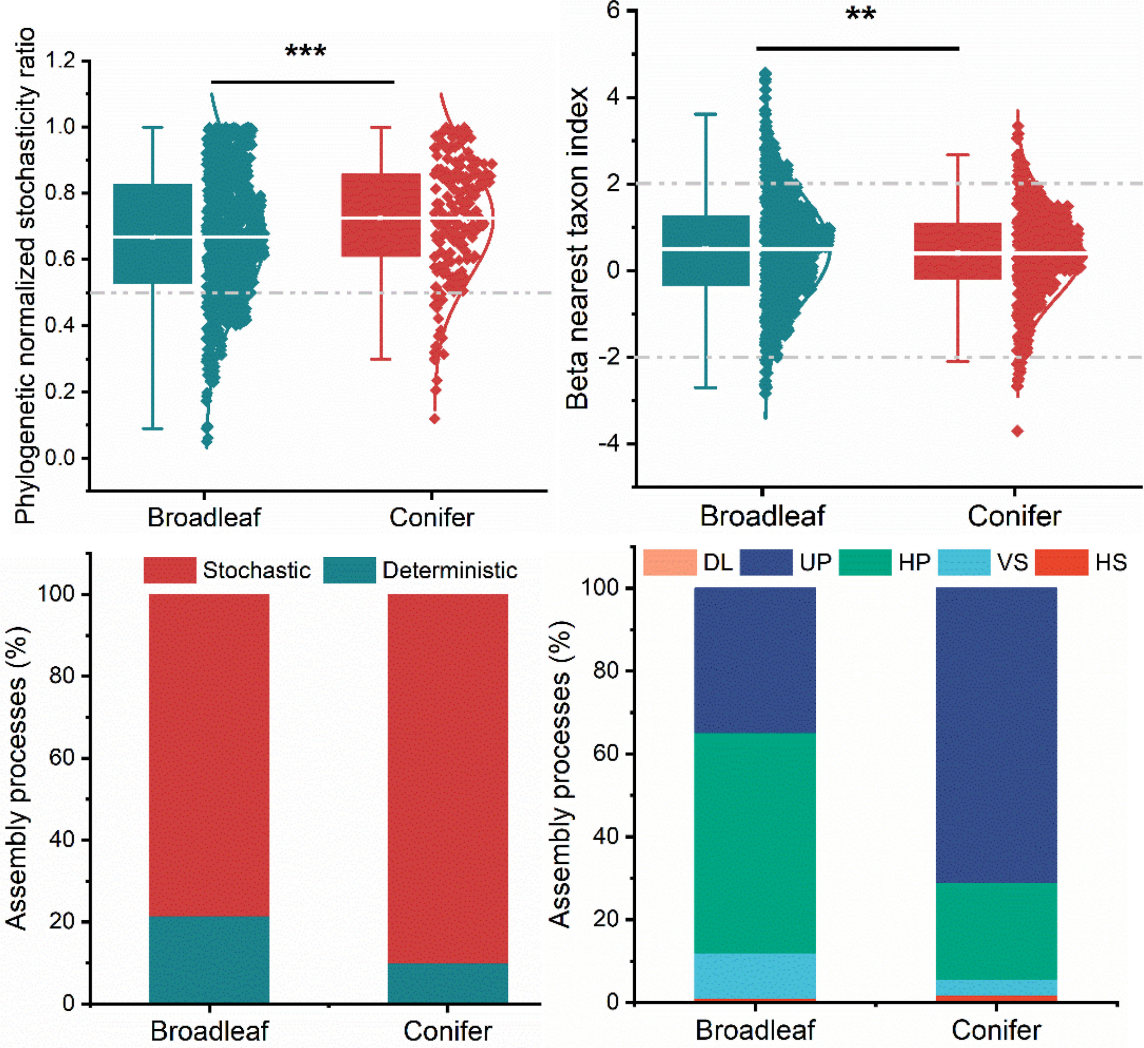
Deterministic vs. stochastic assembly processes in broadleaf and coniferous trees based on phylogenetic normalized stochasticity ratio (pNST) and beta nearest taxon index (βNTI). Stochastic processes: DL, dispersal limitation; UP, undominated processes; HP, homogenizing dispersal; deterministic processes: VS, variable selection; HS, homogeneous selection. The non-dominant processes (|RCbray|<0.95) were drift and diversification *P* < 0.01 = **, *P* < 0.001 = ***.

Analysis using the phylogenetic normalized stochasticity ratio (pNST) and beta nearest taxon index (βNTI) showed that the assembly processes of fungal plant pathogens were highly dominated by stochastic processes (**Figure 4**). However, the contribution of stochasticity of fungal plant pathogens in broadleaf tree species was significantly lower than that in coniferous tree species. Based on βNTI, the stochastic assembly processes comprised mainly of undominated processes (UP) or ecological drift, homogenizing dispersal (HP). However, the patterns are different in broadleaf and coniferous trees. The homogenizing dispersal process dominated the stochastic assembly processes in broadleaf tree species and ecological drift in coniferous tree species. The deterministic assembly processes contributed more in broadleaf trees (~20% estimated by pNST and ~10% by βNTI) as compared to coniferous trees (5–10%) (**Figure 4**). Deterministic assembly processes were dominated by variable selection (VS) with a small contribution of homogeneous selection (HS). We further calculated the normalized stochasticity ratio based on the taxonomic turnover (tNST). High tNST value was observed in broadleaved group (*P*<0.05, **Supplementary Figure S3A**). Based on the tNST value, broadleaf and coniferous trees revealed 36.5% and 28.4% proportions of stochasticity, respectively (**Supplementary Figure S3B**).

### Factors determining fungal plant pathogenic community composition

**Figure 5:**
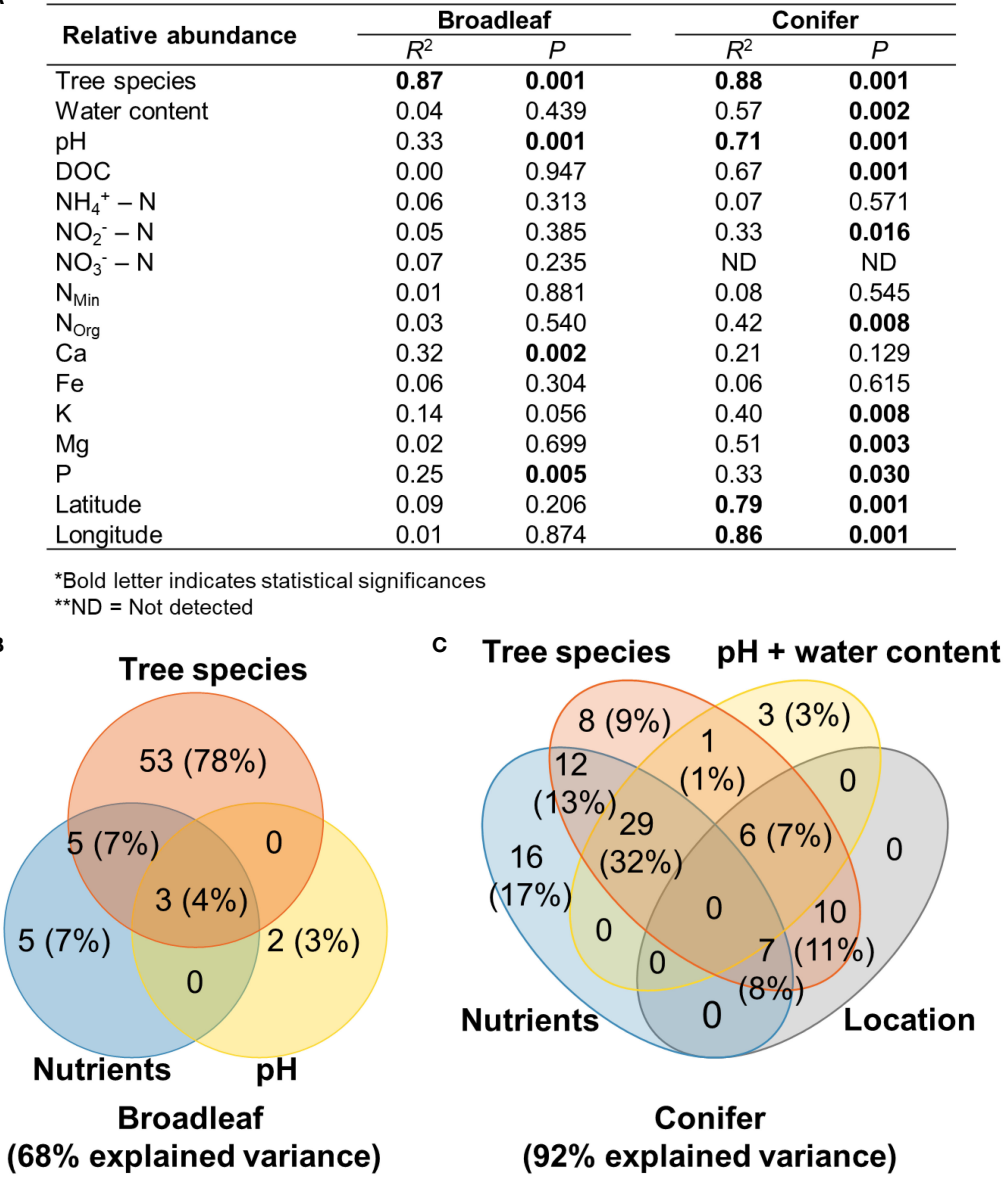
Goodness-of-fit statistics (*R* ^2^) of environmental variables fitted to NMDS ordination of fungal plant pathogenic community based on relative abundance data and Bray–Curtis distance measure **(A)**, Venn diagrams showing the contributions of the factors shaping fungal plant pathogenic community **(B, C)**. Nutrients evaluated in the analysis of broadleaf trees were Ca and P. Nutrients evaluated in the analysis of coniferous trees were DOC, ${\text{NO}}_{2}^{-}$ , N_org_, K, Mg, and P. The number and percentage in the parentheses in the Venn diagram indicate the explained variance and its percentage in the total explainable variance. *Bold letter indicates statistical significance, ** ND, Not detected.

Based on relative abundance data, the fungal plant pathogenic community compositions in broadleaf and coniferous tree species were shaped mainly by tree species identity (*R^2^* = 0.87–0.88, *P* = 0.001) (**Figure 5**). In coniferous tree species, the pH, latitude, and longitude of each tree individual were additional primary factors that shaped fungal plant pathogenic community composition (*R^2^* = 0.71–0.86, *P* = 0.001). Leaf nutrients such as C, N, K, Mg, and P also significantly correlated with fungal plant pathogenic community composition in coniferous tree species. (**Figures 5**). In broadleaf tree species, we also detected correspondence of the fungal plant pathogenic community composition with pH, Ca, and P content (**Figures 5**). Similar pattern of the factors determining the fungal plant pathogenic community compositions was found on the presence/absence data. The tree species identity (*R^2^* = 0.83–0.91, *P* = 0.001) mainly shaped the fungal plant pathogenic community compositions in broadleaf and coniferous tree species (**Supplementary Figure S4**). Leaf/needle water content, pH, latitude, and longitude as well as leaf nutrients (C, N, K, Mg, and P) were also important factors that shaped fungal plant pathogenic community composition (*R^2^* = 0.35–0.91, *P* = 0.024–0.001).

All studied factors that significantly corresponded with the fungal plant pathogenic community composition were used in the variation partitioning analysis. These factors explained 68% and 92% of variation in relative abundance data of fungal plant pathogenic community compositions of broadleaf and coniferous tree species, respectively (**Figures 5B, C**). In broadleaf trees, tree species alone explained the highest variation in fungal plant pathogenic community composition (78% of the total explainable variance). Nutrients alone explained 7% of the variation in fungal plant pathogenic community composition in broadleaf trees and their combining effect with tree species explained 7%. In coniferous trees, nutrient abundance alone explained the highest variation (17% of the total explainable variance), followed by tree species and pH/water content. Combining effect of nutrients and tree species explained 13% of the variation in fungal pathogenic community composition in coniferous trees. Location (latitude and longitude) alone did not explain the variation in the fungal plant pathogenic community in coniferous trees. Tree species identity, nutrients, pH/water content, and their combined effects explained more than 70% of the explainable variance in both tree types.

While tree species, nutrients, pH, water content, and location explained large proportion of variation in fungal plant pathogen community compositions based on relative abundance data, they explain 25% and 41% of those based on presence/absence data in broadleaf and coniferous tree species, respectively (**Supplementary Figures S4B, C**). Nevertheless, similar pattern of variation partitioning was found. In broadleaf trees, tree species alone explained the highest variation in fungal plant pathogenic community composition (68% of the total explainable variance), followed by nutrients (4%). Combining effect of nutrients and tree species explained 8% of variation in fungal plant pathogenic community composition. In coniferous trees, nutrient alone also explained the highest variation (12% of the total explainable variance), followed by tree species and pH/water content. Combining effect of nutrients and tree species explained 5% of the variation in fungal pathogenic community composition in coniferous trees. Location (latitude and longitude) alone did not explain the variation in the fungal plant pathogenic community in both broadleaf and coniferous trees.

### Different co-occurrence network patterns in broadleaf and coniferous trees

**Figure 6:**
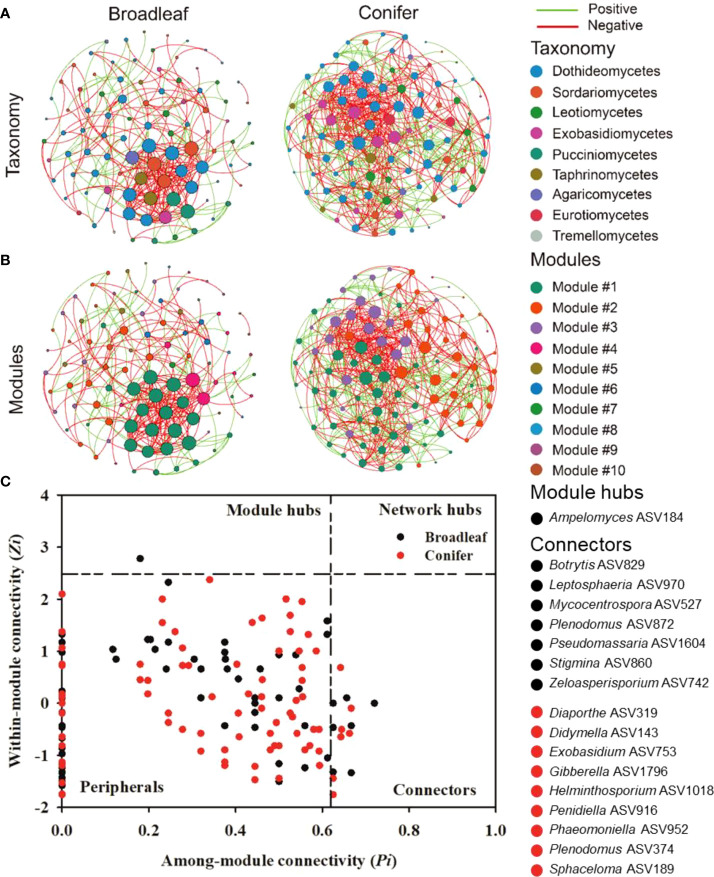
Taxonomic **(A)**, modular networks **(B)**, and topological roles of each ASVs in the fungal plant pathogenic co-occurrence networks **(C)** in broadleaf and coniferous tree species. The green (co-presence, positive) and red links (mutual exclusion, negative) in the co-occurrence networks represent significant Spearman’s correlations (*P*<0.05). Each node in Fig. 6a and b represents one ASV. The size of each node is proportional to the degree. The node color indicates the corresponding taxonomic assignment at the class **(A)** and modular level **(B)**. The nodes in Fig. 6c with *Zi* > 2.5 but *Pi*< 0.62 are identified as module hubs, and those with *Pi* > 0.62 but *Zi*< 2.5 represent connectors. The network hubs are characterized with *Zi* > 2.5 and *Pi* > 0.62, and the peripherals are characterized with *Zi*< 2.5 and *Pi*< 0.62 according to Olesen et al., 2007. Nodes in broadleaf 10 (4 big modules), conifer 4 (3 big modules > 10% of the notes) modules.

To evaluate the interactions among fungal plant pathogenic taxa and the co-occurrence network patterns of both tree types, two ecological networks for broadleaf and coniferous tree species were constructed (**Figure 6**). The main topological parameters in the empirical network were higher than in the random network, which implied a nonrandom pattern. The fungal plant pathogenic network in coniferous trees was more complex than those in broadleaf trees, revealing a lower average path distance, average clustering coefficient, and modularity but higher average degree (**Supplementary Table S3**). Notably, the percentage of negative links (63.5%) in the coniferous network was lower than in broadleaf trees (75.5%). Furthermore, we detected 10 and four modules (functional units or subcommunities) in the networks of broadleaf and coniferous trees, respectively. However, the numbers of large modules (modules with more than 10% notes) were similar in both tree types (4 large modules in broadleaf and 3 large modules in coniferous trees, **Figure 6**).

Based on *Zi* and *Pi* tests, the potential topological roles of the fungal plant pathogenic taxa were explored (**Figure 6C**). One node belonging to *Ampelomyces* ASV184 was identified as a module hub in broadleaf trees. We detected seven and nine connectors belonging to diverse fungal plant pathogenic genera in the broadleaf and coniferous networks, respectively. The keystone species (module hub/connectors) were distinct between the tree types. The fungal plant pathogenic module hub, *Ampelomyces* ASV184, was classified as a specialist and detected solely in *C. betulus*. Conversely, connectors were distributed across different tree species. Among them, four connectors were classified as fungal plant pathogenic generalists, including *Botrytis* ASV829, *Gibberella* ASV1796, *Pseudomassaria* ASV1604, and *Sphaceloma* ASV189.

### Fate of fungal plant pathogens

**Figure 7:**
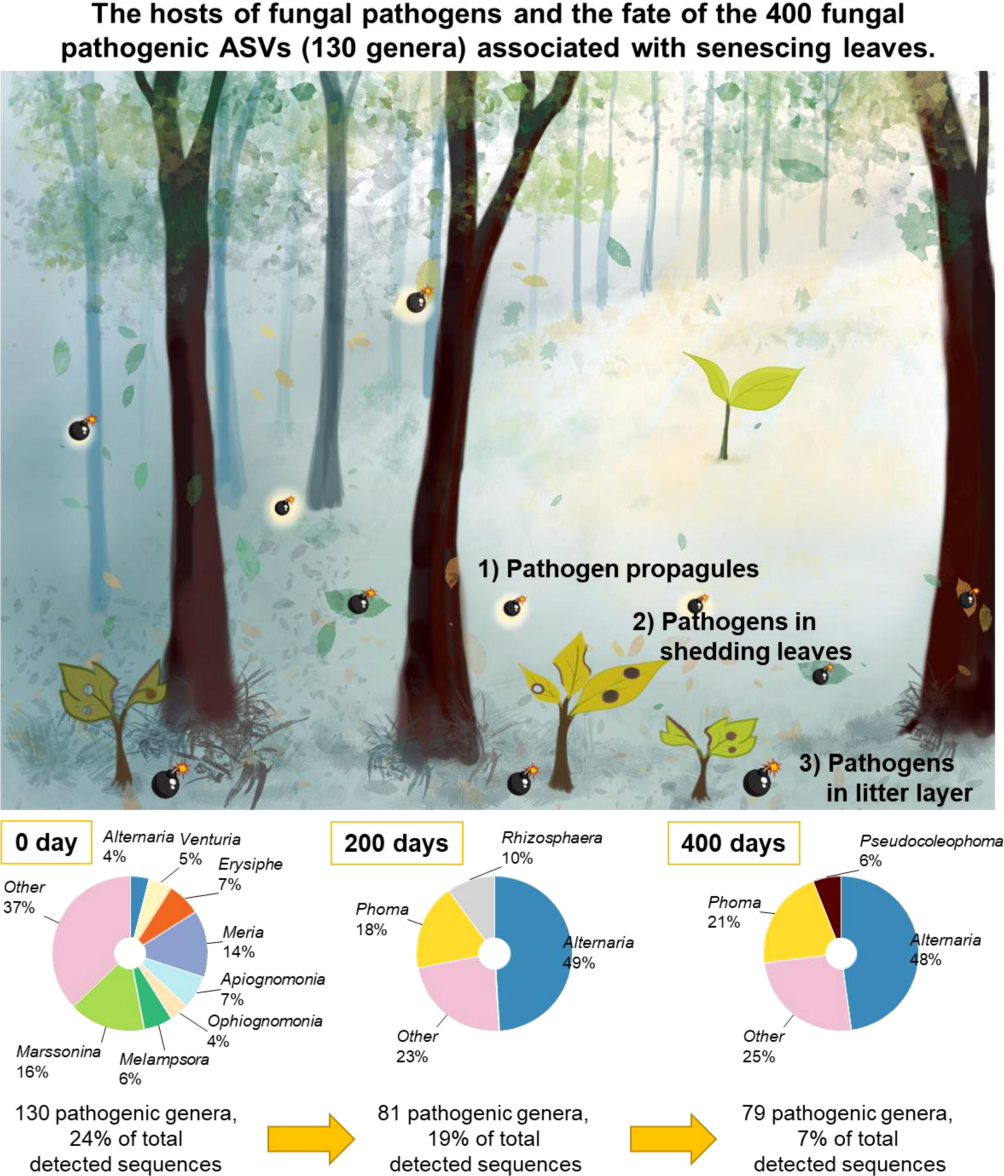
The fate of the 400 fungal pathogenic ASVs associated with senescing leaves and needles. Leaf-associated fungal pathogens at 200 and 400 days were characterized by fungal internal transcribed spacer (ITS)-based amplicon sequencing on the Illumina MiSeq sequencing with the same bioinformatics.

After 200 days of decomposition, 81 out of 130 plant pathogenic fungal genera were detected in the decomposing leaves and needles (**Figure 7**). The proportion of *Alternaria* increased from 4% to 49% of the sequence reads of the considered plant pathogens. *Phoma* (18%) and *Rhizosphaera* (10%) co-dominated along the plant pathogenic fungal genera along with *Alternaria*. After 400 days of decomposition, 79 out of 130 plant pathogenic genera continued to colonize the decomposing leaves and needles (**Figure 7**). *Alternaria* (48%) and *Phoma* (21%) hyperdominated the sequence read of the 79 plant pathogens.

## Discussion

### Pathobiome of 12 common temperate tree species

We successfully accessed the pathobiomes in senescing leaves and needles of the 12 common temperate tree species, which allowed us to compare fungal plant pathogen load among different tree species. Our current study revealed diverse and tree species distinct fungal plant pathogens associated with senescing leaves and needles of the 12 common temperate tree species, which is appropriate to be considered as the database for foliar fungal pathogens in Thuringia forest (**Supplementary Tables S1, S4**). With the aid of the high-resolution molecular approach (Ilumina MiSeq) used in this study, our understanding of foliar fungal pathobiome communities was significantly extended. Despite the fact that our experiment focused at the local scale, we detected highly diverse and abundant fungal plant pathogens associated with senescing leaves and needles of 12 temperate tree species. The majority of these fungal plant pathogens were species-specific. In *F. sylvatica*, the fungal plant pathogenic generalist *Mycosphaerella* ASV14 (UNITE name: *Mycosphaerella punctiformis*) was highly abundant. In *A. pseudoplantanus*, diverse fungal plant pathogenic generalists were detected, including *Mycosphaerella* ASV14. *Mycosphaerella* spp. have been reported to cause leaf spot in different tree species, including *Acer* (Funk and Dorworth, 1988), *Fraxinus* (Wolf and Davidson, 1941), *Fagus* (Burke et al., 2020), and *Tilia* (Bernadovičová and Ivanová, 2008). In *C. butulus*, we also detected a high richness of fungal plant pathogenic generalists and high relative abundances of *Mycosphaerella* ASV14 and the fungal plant pathogenic specialist *Erysiphe* ASV42 (UNITE name: *Erysiphe arcuata*), which has previously been reported as leaf disease pathogen in *C. butulus* (Chinan and Mânzu, 2021). Apart from the previously described hosts of foliar plant pathogens (airborne pathogen propagules and spore) (Bayandala et al., 2016), we suggest that fungal plant pathogens inhabiting senescing leaves can act as an agent to cause foliar disease in seedlings which is in line with a previous study on contribution of leaf litter on *Mycosphaerella* leaf disease (Sánchez Márquez et al., 2011). In **Figure 7**, we summarize the aforementioned hosts of foliar pathogens, which the mature tree uses to regulate seedling density. Furthermore, we preliminary investigated the fate of these plant pathogens in senescing leaves and needles during the decomposition process (**Figure 7**). In senescing leaves and needles, fungal pathogenic community composition was dominated by diverse fungal genera, including *Marssonina*, *Meria*, *Erysiphe*, *Apiognomonia*, and *Melampsora*. After 200 days of leaf decomposition, the high diversity and relative abundance of the previously detected foliar fungal plant pathogenic community are maintained. Nevertheless, the relative abundance of the foliar pathogens strongly declines after 400 days of decomposition. *Alternaria* is hyper-dominant with some contributions of *Phoma* at 200 and 400 days. Thus, decomposing leaf litter can be an important agent causing foliar disease in seedlings and even saplings over seasons.

### Pathobiome community assembly is highly determined by stochastic processes

Understanding the process governing fungal plant pathogenic community assembly is important for identifying driving factors. Some studies have focused on environmental filtering and seek factors to describe the community, but the community assembly may be largely explained by stochastic processes (Abrego, 2021). This study demonstrates that the fungal pathobiome associated with leaves and needles of 12 common temperate tree species are determined mainly by stochastic processes based on pNST, specifically homogenizing dispersal and undominated processes (ecological drift). Based on tNST, deterministic processes play more important role as compared with stochastic processes. The controversial results of pNST and tNST were also demonstrated in a previous study based on bacterial 16S rRNA amplicon (Tai et al., 2020). This may be due to the calculation of the microbial attributes from different dimensions of diversity (including taxonomic, phylogenetic, functional, etc.). Thus, the results should be interpreted with caution. Nevertheless, stochasticity based on the tNST also reveals substantial proportion in governing fungal plant pathogenic community assembly in both broadleaf (36.5%) and coniferous trees (28.4%). Tree species is the main factor that shapes fungal plant pathogenic communities in both broadleaf and coniferous tree species. The results of negative networks and stochasticity suggest that the systems in both tree types follow the colonization–extinction stochasticity assumption (unpredictability in arrival and establishment of different species) (Abrego, 2021). This implies that the fungal pathobiome colonizing the leaf habitat exhibits unpredictable systematic variation in species, which can be driven by the colonization–competition trade-off (Tilman, 1994; Abrego, 2021). We determined the contribution of deterministic processes, mainly by variable selection (VS). We found that pH and P level significantly correspond with fungal plant pathogenic community compositions in both tree types. Other important factors in broadleaf or coniferous trees are water content, C, N, and other leaf nutrients. Tree species can also determine leaf nutrients such as Ca, P, and N. In this study, we found the importance of both nutrients alone as well as their combined effect with tree species in explaining the variation in fungal plant pathogenic community. These factors were previously reported to shape microbial community composition associated with decomposing leaf of European beech in Germany (Purahong et al., 2016). These microbial macronutrients are important for microbial growth, reproductivity, and activity (Prescott et al., 1999; Purahong et al., 2015; Purahong et al., 2016).

## Conclusion

Our study is the first to investigate community assembly, networks, and the complete taxonomy of foliar fungal pathobiome, which different tree species may use for inter- and intraspecific competition in mixed temperate forests. Shedding the foliar fungal pathogens with senescing leaf seems to be an effective strategy as it can be repeated over years and the healthy mature tree would not be affected much by their own foliar fungal pathogens. Future studies should focus on the fate of fungal plant pathogens (colonization–extinction dynamics) and how their interaction change during the leaf decomposition process.

## Data availability statement

The datasets presented in this study can be found in online repositories. The names of the repository/repositories and accession number(s) can be found in the article/**Supplementary Material**.

## Author contributions

WP and E-DS conceived and designed the study. BT, WP, E-DS, and SW collected samples and metadata. WP and FB contributed reagents and laboratory equipment. BT, WP, and SW led the DNA analysis. SW led bioinformatics. BT, LJ, and WP led the microbial taxonomy and data analyses. SS, GG, A-SL, and EA led the physicochemical analyses. BT, LJ, and WP wrote the manuscript. MN and WP supervised BT. MN, E-DS, PL, and FB reviewed and gave comments and suggestions for manuscript. All authors contributed to the article and approved the submitted version.

## Funding

This work was partially funded by the internal research budget of WP to Department of Soil Ecology, UFZ-Helmholtz Centre for Environmental Research.

## Acknowledgments

Community composition data were computed at the High-Performance Computing (HPC) Cluster EVE, a joint effort of both the Helmholtz Centre for Environmental Research - UFZ and the German Centre for Integrative Biodiversity Research (iDiv) Halle-Jena-Leipzig. We thank Beatrix Schnabel and Melanie Günther for their help with Illumina sequencing.

## Conflict of interest

The authors declare that the research was conducted in the absence of any commercial or financial relationships that could be construed as a potential conflict of interest.

## Publisher’s note

All claims expressed in this article are solely those of the authors and do not necessarily represent those of their affiliated organizations, or those of the publisher, the editors and the reviewers. Any product that may be evaluated in this article, or claim that may be made by its manufacturer, is not guaranteed or endorsed by the publisher.
