# Supplementary material for: A poisoned apple: First insights into community assembly and networks of the fungal pathobiome of healthy-looking senescing leaves of temperate trees in mixed forest ecosystem
Source: Front Plant Sci. 2022 Nov 3;13:968218. doi: 10.3389/fpls.2022.968218 (PMC9669904; doi:10.3389/fpls.2022.968218)

*Supplementary Material*

# A poisoned apple: First insights into community assembly and networks of the fungal pathobiome of healthy-looking senescing leaves of temperate trees in mixed forest ecosystem.

**Benjawan Tanunchai[1,2¶], Li Ji[1,3¶], Simon Andreas Schroeter[4], Sara Fareed Mohamed Wahdan[1,5], Panadda Larpkern[6], Ann-Sophie Lehnert[4], Eliane Gomes Alves[4], Gerd Gleixner[4], Ernst-Detlef Schulze[4], Matthias Noll[2,7], François Buscot[1,8*], Witoon Purahong[1*]**

[1]UFZ-Helmholtz Centre for Environmental Research, Department of Soil Ecology, Theodor-Lieser-Str. 4, 06120 Halle (Saale), Germany

[2]Department of Biology, University of Bayreuth, Bayreuth, Germany

[3]Key Laboratory of Sustainable Forest Ecosystem Management-Ministry of Education, School of Forestry, Northeast Forestry University, 150040 Harbin, P.R. China

[4]Max Planck Institute for Biogeochemistry, Biogeochemical Processes Department, Hans-Knöll-Str. 10, 07745 Jena, Germany.

[5]Botany Department, Faculty of Science, Suez Canal University, 41522 Ismailia, Egypt

[6]Bodhivijjalaya College, Srinakharinwirot University, Nakhon Nayok, Thailand. (panaddal@g.swu.ac.th)

[7]Institute of Bioanalysis, Department of Applied Science, University of Coburg, Coburg, Germany

[8]German Centre for Integrative Biodiversity Research (iDiv), Halle-Jena-Leipzig, Deutscher Platz 5e, 04103 Leipzig, Germany

*** Correspondence:** Corresponding Author witoon.purahong@ufz.de

**Physiochemical analyses**

To obtain water-leachable components, senescing leaf and needle samples were shaken in 30 mL milliQ water for 1 h in falcon tubes, centrifuged for 5 min at 3500 rpm, decanted, and filtered. The remaining leaf/needle material was dried for two weeks at 40 °C to determine dry weight, which was used as reference for all subsequent qualifications. Leachate pH was determined using pH paper with a scale precision of 0.2 units. TN was analyzed using a sum parameter analyzer with high temperature combustion and chemiluminescence detection (Mitsubishi TN-100; a1 envirosciences, Düsseldorf, Germany). All samples were measured as triplicates. $N_{org}$ was calculated as the difference: $N_{org}$ = TN–$N_{Min}$. For $N_{Min}$ quantification, a flow injection analyzer (Quikchem QC85S5; Lachat Instruments, Hach Company, Loveland CO, USA) was used with corresponding manifolds to measure ammonium nitrogen $N_{NH_4^+}$, nitrite nitrogen $N_{NO_2^-}$, and nitrate- plus nitrite nitrogen $N_{NO_3^-+NO_2^-}$ content. $N_{NH_4^+}$ was determined by the gas diffusion method. $N_{NO_3^-}$ was reduced to nitrite using a cadmium column in the manifold prior to the chemical reaction to form an azo dye. The nitrate reduced by cadmium and the nitrite originally present in the sample were analyzed using the Griess reaction by diazotization with sulfanilamide and coupling with N-(1-naphthyl) ethylenediamine dihydrochloride. The deep pink color of the resulting dye was measured at λ = 520 nm. $N_{NO_2^-}$ alone was determined after the same reaction, without using a cadmium column. DOC was quantified as non-purgeable organic carbon (NPOC) with a sum parameter analyzer using high-temperature combustion and infrared detection (vario TOC cube, Elementar Analysensysteme GmbH, Langenselbold, Germany). Each sample was measured as triplicate. A sample volume of 200 µL each was automatically injected into the ash finger of the combustion tube which contains platinum as catalyst. The samples were combusted at 850°C in synthetic air, a hydrocarbon-free mixture of nitrogen and oxygen. After removing moisture from the

combustion gas, NPOC was quantified by IR detection of $CO_2$ formed from the organic carbon compounds in the sample.

The determination of nutrient content, Ca, Fe, K, Mg, and P of leaves and needles followed two processes. First, the sample digestion, in which 100 mg of sample material were submitted to a microwave-assisted high-pressure digestion (Multiwave 3000, Anton Paar, Graz, Austria) at a maximum microwave power of 1200 W and a maximum pressure of 60 bar after addition of 3 – 5 mL 65% $HNO_3$, supra-pur, (Merck, Darmstadt, Germany). A rotor 8SXF100 with reaction vessels made of TFM (tetrafluor-modified polytetrafluoroethylene) was used. Overall digestion time was 50 min, including 20 min of cooling at zero microwave power. A blank, consisting of nitric acid only was run to check for possible contamination of reagents and vessels. After accomplishment of digestion, the solutions were filtered and transferred to 50 mL PE vessels which were filled to the mark with ultrapure water (Millipore, Eschborn, Germany). Secondly, the sample solution analyses were carried out using inductively coupled plasma–optical emission spectrometry (ICP-OES) “Arcos” (Spectro, Kleve, Germany) equipped with a 27.12 MHz free-running LDMOS generator and ORCA optical system. A three-point-calibration based on single-element standards issued by Merck, Darmstadt, Germany, was carried out at the following concentration levels: 10, 50, 100 mg/L for Ca, K, Mg, P and 0.5, 2.5 and 5 mg/L for Fe, respectively.

## R scripts for statistical analyses

### *Specialist/generalist classification*

```
setwd("C:\\Initial_leaves_fungi")

install.packages("vegan")

install.packages("spaa")
```

```
install.packages("devtools")

devtools::install_github("GuillemSalazar/EcolUtils")

library(EcolUtils)

library(RCurl)

comm.tab<-
read.table("Pathogen_spe_gen.txt",text=x,sep="\t",row.names=1,header=TRUE,comment.char="@")

comm.tab<-t(comm.tab[,1:60])

comm.tab<-comm.tab[,which(colSums(comm.tab)>0)]

res<-spec.gen(comm.tab,n=100)

comm.tab.bin<-ceiling(comm.tab/max(comm.tab))

colors <- c("dodgerblue4", "gray47", "orangered3")

plot(colSums(comm.tab),colSums(comm.tab.bin)/dim(comm.tab.bin)[1], family = "ArialMT" ,col=
colors[res$sign], pch=19,log="x",xlab="Abundance",ylab="Occurrence", cex.lab = 1.5, cex.axis = 1,
cex = 1)

legend("topleft",levels(res$sign), col=colors,pch=19,inset=0.01,cex=1.5)

install.packages("xlsx")

library("xlsx")

write.xlsx(res, file = "Pathogen_spec_gen.xlsx", sheetName = "Specialist_Generalist",
```

```
col.names = TRUE, row.names = TRUE, append = FALSE)
```

***Goodness-of-fit statistic and variance partitioning analyses***

```
setwd("C:\\Initial_leaves_fungi")

library(vegan)

FUNA=read.csv("PP_presence_absence_factors_C.csv", header=T)

fix(FUNA)

row.names(FUNA)=FUNA$Plant_sample

speciesf=  FUNA[,21 :420]

environmentf=FUNA[,2:20]

fix(speciesf)

fix(environmentf)

FUNA.nmds<-metaMDS( speciesf ,distance="jaccard", k=2, autotransform=FALSE,trace = FALSE)

FUNA.nmds

plot(FUNA.nmds)

fit_FUNA<-envfit(FUNA.nmds, environmentf, perm=999,na.rm = TRUE)

fit_FUNA

modall <- varpart(speciesf, ~ TreeID, ~ TOC + Norg + K + Mg + P , ~ pH + Water_content, ~Lat + Long,  data=environmentf)

modall

plot(modall)
```

**Table S1.** Average relative abundance of fungal plant pathogens detected in senescing leaves and needles of 12 temperate tree species (please see in a separate excel file).

**Table S2.** Comparisons between fungal plant pathogenic community compositions associated with 12 temperate tree species using analysis of similarities (ANOSIM) and non-parametric multivariate analysis of variance (NPMANOVA) based on (a) relative abundance data and the Bray-Curtis distance measure and (b) presence/absence data and the Jaccard distance measure.

(a) ANOSIM and NPMANOVA based on relative abundance data and the Bray-Curtis distance measure.

| Comparison | NPMANOVA | ANOSIM |
|---|---|---|
| *A. pseudoplatanus vs. F. sylvatica* | 4.49** | 0.54** |
| *A. pseudoplatanus vs. P. menziesii* | 4.63** | 0.99** |
| *A. pseudoplatanus vs. Q. robur* | 4.42** | 0.94** |
| *A. pseudoplatanus vs. F. excelsior* | 3.31* | 0.74* |
| *A. pseudoplatanus vs. P. abies* | 5.50** | 1.00** |
| *A. pseudoplatanus vs. C. betulus* | 5.81** | 0.59** |
| *A. pseudoplatanus vs. P. avium* | 1.78* | 0.28* |
| *A. pseudoplatanus vs. P. sylvestris* | 7.75** | 1.00** |
| *A. pseudoplatanus vs. L. decidua* | 9.43** | 0.98** |
| *A. pseudoplatanus vs. T. cordata* | 4.11** | 0.65** |
| *A. pseudoplatanus vs. P. Hybrid* | 9.29** | 1.00** |
| *F. sylvatica vs. P. menziesii* | 10.49** | 1.00** |
| *F. sylvatica vs. Q. robur* | 8.89** | 0.85** |
| *F. sylvatica vs. F. excelsior* | 8.77** | 0.88** |
| *F. sylvatica vs. P. abies* | 12.93** | 1.00** |
| *F. sylvatica vs. C. betulus* | 16.63** | 0.99** |
| *F. sylvatica vs. P. avium* | 5.07** | 0.60** |
| *F. sylvatica vs. P. sylvestris* | 20.97** | 1.00** |
| *F. sylvatica vs. L. decidua* | 34.76** | 1.00** |
| *F. sylvatica vs. T. cordata* | 9.77** | 0.82** |
| *F. sylvatica vs. P. Hybrid* | 29.93** | 1.00* |
| *P. menziesii vs. Q. robur* | 5.32** | 1.00** |
| *P. menziesii vs. F. excelsior* | 4.84** | 1.00** |
| *P. menziesii vs. P. abies* | 4.36** | 0.94** |
| *P. menziesii vs. C. betulus* | 11.10** | 1.00** |

| Comparison | NPMANOVA | ANOSIM |
| --- | --- | --- |
| *P. menziesii vs. P. avium* | 3.78** | 0.93** |
| *P. menziesii vs. P. sylvestris* | 7.17** | 0.98** |
| *P. menziesii vs. L. decidua* | 10.22** | 1.00** |
| *P. menziesii vs. T. cordata* | 7.64** | 1.00** |
| *P. menziesii vs. P. Hybrid* | 10.28** | 1.00** |
| *Q. robur vs. F. excelsior* | 4.42** | 0.95** |
| *Q. robur vs. P. abies* | 6.31** | 1.00** |
| *Q. robur vs. C. betulus* | 10.70** | 0.94** |
| *Q. robur vs. P. avium* | 3.46** | 0.80** |
| *Q. robur vs. P. sylvestris* | 8.98** | 1.00** |
| *Q. robur vs. L. decidua* | 11.52** | 1.00** |
| *Q. robur vs. T. cordata* | 7.26** | 1.00** |
| *Q. robur vs. P. Hybrid* | 10.78** | 1.00** |
| *F. excelsior vs. P. abies* | 5.58** | 1.00** |
| *F. excelsior vs. C. betulus* | 8.38** | 0.83** |
| *F. excelsior vs. P. avium* | 2.34* | 0.52** |
| *F. excelsior vs. P. sylvestris* | 8.04** | 1.00** |
| *F. excelsior vs. L. decidua* | 9.88** | 0.99** |
| *F. excelsior vs. T. cordata* | 5.08** | 0.78** |
| *F. excelsior vs. P. Hybrid* | 9.36** | 1.00** |
| *P. abies vs. C. betulus* | 13.71** | 1.00** |
| *P. abies vs. P. avium* | 4.26** | 0.93** |
| *P. abies vs. P. sylvestris* | 7.84** | 1.00** |
| *P. abies vs. L. decidua* | 12.03** | 1.00** |
| *P. abies vs. T. cordata* | 9.07** | 1.00** |
| *P. abies vs. P. Hybrid* | 12.00** | 1.00** |
| *C. betulus vs. P. avium* | 5.51** | 0.58* |
| *C. betulus vs. P. sylvestris* | 23.22** | 1.00** |
| *C. betulus vs. L. decidua* | 39.78** | 1.00** |
| *C. betulus vs. T. cordata* | 12.28** | 0.92** |
| *C. betulus vs. P. Hybrid* | 33.98** | 1.00** |
| *P. avium vs. P. sylvestris* | 6.58** | 1.00** |
| *P. avium vs. L. decidua* | 7.90** | 0.96** |
| *P. avium vs. T. cordata* | 3.98** | 0.61** |
| *P. avium vs. P. Hybrid* | 7.87** | 1.00** |
| *P. sylvestris vs. L. decidua* | 18.65** | 1.00** |
| *P. sylvestris vs. T. cordata* | 13.69** | 1.00** |
| *P. sylvestris vs. P. Hybrid* | 18.63** | 1.00** |
| *L. decidua vs. T. cordata* | 19.25* | 1.00** |
| *L. decidua vs. P. Hybrid* | 30.73** | 1.00** |
| *T. cordata vs. P. Hybrid* | 17.11** | 1.00** |

(b) ANOSIM and NPMANOVA based on presence/absence data and the Jaccard distance measure.

| Comparison | NPMANOVA | ANOSIM |
|---|---|---|
| *A. pseudoplatanus vs. F. sylvatica* | 2.90** | 0.74** |
| *A. pseudoplatanus vs. P. menziesii* | 3.87** | 1.00** |
| *A. pseudoplatanus vs. Q. robur* | 2.17** | 0.67** |
| *A. pseudoplatanus vs. F. excelsior* | 2.65** | 0.82** |
| *A. pseudoplatanus vs. P. abies* | 6.08** | 1.00** |
| *A. pseudoplatanus vs. C. betulus* | 3.12** | 0.70** |
| *A. pseudoplatanus vs. P. avium* | 2.13** | 0.53** |
| *A. pseudoplatanus vs. P. sylvestris* | 6.89** | 1.00** |
| *A. pseudoplatanus vs. L. decidua* | 4.20** | 1.00** |
| *A. pseudoplatanus vs. T. cordata* | 2.11** | 0.62** |
| *A. pseudoplatanus vs. P. Hybrid* | 5.30** | 1.00** |
| *F. sylvatica vs. P. menziesii* | 4.95** | 1.00** |
| *F. sylvatica vs. Q. robur* | 3.19** | 0.84** |
| *F. sylvatica vs. F. excelsior* | 4.50** | 1.00** |
| *F. sylvatica vs. P. abies* | 8.32** | 1.00** |
| *F. sylvatica vs. C. betulus* | 3.86** | 0.90** |
| *F. sylvatica vs. P. avium* | 3.40** | 0.80** |
| *F. sylvatica vs. P. sylvestris* | 9.94** | 1.00** |
| *F. sylvatica vs. L. decidua* | 5.93** | 1.00** |
| *F. sylvatica vs. T. cordata* | 2.91** | 0.82** |
| *F. sylvatica vs. P. Hybrid* | 7.73** | 1.00** |
| *P. menziesii vs. Q. robur* | 4.07** | 1.00** |
| *P. menziesii vs. F. excelsior* | 4.66** | 1.00** |
| *P. menziesii vs. P. abies* | 3.92** | 0.97** |
| *P. menziesii vs. C. betulus* | 5.51** | 1.00** |
| *P. menziesii vs. P. avium* | 4.11** | 1.00** |
| *P. menziesii vs. P. sylvestris* | 5.21** | 1.00** |
| *P. menziesii vs. L. decidua* | 3.36** | 0.97** |
| *P. menziesii vs. T. cordata* | 4.36** | 1.00** |
| *P. menziesii vs. P. Hybrid* | 6.14** | 1.00** |
| *Q. robur vs. F. excelsior* | 2.38** | 0.72** |
| *Q. robur vs. P. abies* | 6.16** | 1.00** |
| *Q. robur vs. C. betulus* | 3.03** | 0.72** |
| *Q. robur vs. P. avium* | 1.77** | 0.34** |
| *Q. robur vs. P. sylvestris* | 7.23** | 1.00** |
| *Q. robur vs. L. decidua* | 4.61** | 1.00** |
| *Q. robur vs. T. cordata* | 2.09** | 0.61** |
| *Q. robur vs. P. Hybrid* | 5.47** | 1.00** |
| *F. excelsior vs. P. abies* | 6.75** | 1.00** |
| *F. excelsior vs. C. betulus* | 4.43** | 0.91** |

| Comparison | NPMANOVA | ANOSIM |
|---|---|---|
| *F. excelsior vs. P. avium* | 2.39** | 0.60** |
| *F. excelsior vs. P. sylvestris* | 7.87** | 1.00** |
| *F. excelsior vs. L. decidua* | 5.43** | 1.00** |
| *F. excelsior vs. T. cordata* | 2.37** | 0.68** |
| *F. excelsior vs. P. Hybrid* | 5.75** | 0.99** |
| *P. abies vs. C. betulus* | 9.00** | 1.00** |
| *P. abies vs. P. avium* | 6.43** | 1.00** |
| *P. abies vs. P. sylvestris* | 5.93** | 1.00** |
| *P. abies vs. L. decidua* | 5.35** | 1.00** |
| *P. abies vs. T. cordata* | 6.82** | 1.00* |
| *P. abies vs. P. Hybrid* | 8.61** | 1.00** |
| *C. betulus vs. P. avium* | 3.59** | 0.78** |
| *C. betulus vs. P. sylvestris* | 11.05** | 1.00** |
| *C. betulus vs. L. decidua* | 6.47** | 1.00** |
| *C. betulus vs. T. cordata* | 2.87** | 0.71** |
| *C. betulus vs. P. Hybrid* | 8.28** | 1.00** |
| *P. avium vs. P. sylvestris* | 7.26** | 1.00** |
| *P. avium vs. L. decidua* | 4.69** | 1.00** |
| *P. avium vs. T. cordata* | 2.01** | 0.44** |
| *P. avium vs. P. Hybrid* | 5.34** | 0.99** |
| *P. sylvestris vs. L. decidua* | 6.27** | 1.00** |
| *P. sylvestris vs. T. cordata* | 7.98** | 1.00** |
| *P. sylvestris vs. P. Hybrid* | 9.87** | 1.00** |
| *L. decidua vs. T. cordata* | 5.13* | 1.00** |
| *L. decidua vs. P. Hybrid* | 7.09** | 1.00** |
| *T. cordata vs. P. Hybrid* | 5.71** | 0.97** |

**Table S3** Topological characteristics of the ecological networks of the plant pathogen in broadleaved and coniferous tree species.

| Network features | | Broadleaved tree species | Coniferous tree species |
|---|---|---|---|
| Empirical network | Number of nodes | 96 | 103 |
| | Number of links | 286 | 498 |
| | $R^2$ of power-law | 0.46 | 0.29 |
| | Number of positive correlations | 70 (24.5%) | 182 (36.5%) |
| | Number of negative correlations | 216 (75.5%) | 316 (63.5%) |
| | Average degree (avgK) | 5.96 | 9.67 |
| | Average clustering coefficient (avgCC) | 0.29 | 0.22 |
| | Average path distance (GD) | 3.23 | 2.46 |
| | Modularity | 0.45 | 0.33 |
| Random network | avgCC ± SD | 0.16 ± 0.02 | 0.13 ± 0.01 |
| | GD ± SD | 2.74 ± 0.05 | 2.33 ± 0.02 |
| | Modularity ± SD | 0.32 ± 0.01 | 0.24 ± 0.01 |

**Table S4** Latitude and longitude of each tree replicate (please see in a separate excel file).

**Figure S1** NMDS ordinations of plant pathogenic community compositions based on presence/absence data and Jaccard distance similarity.

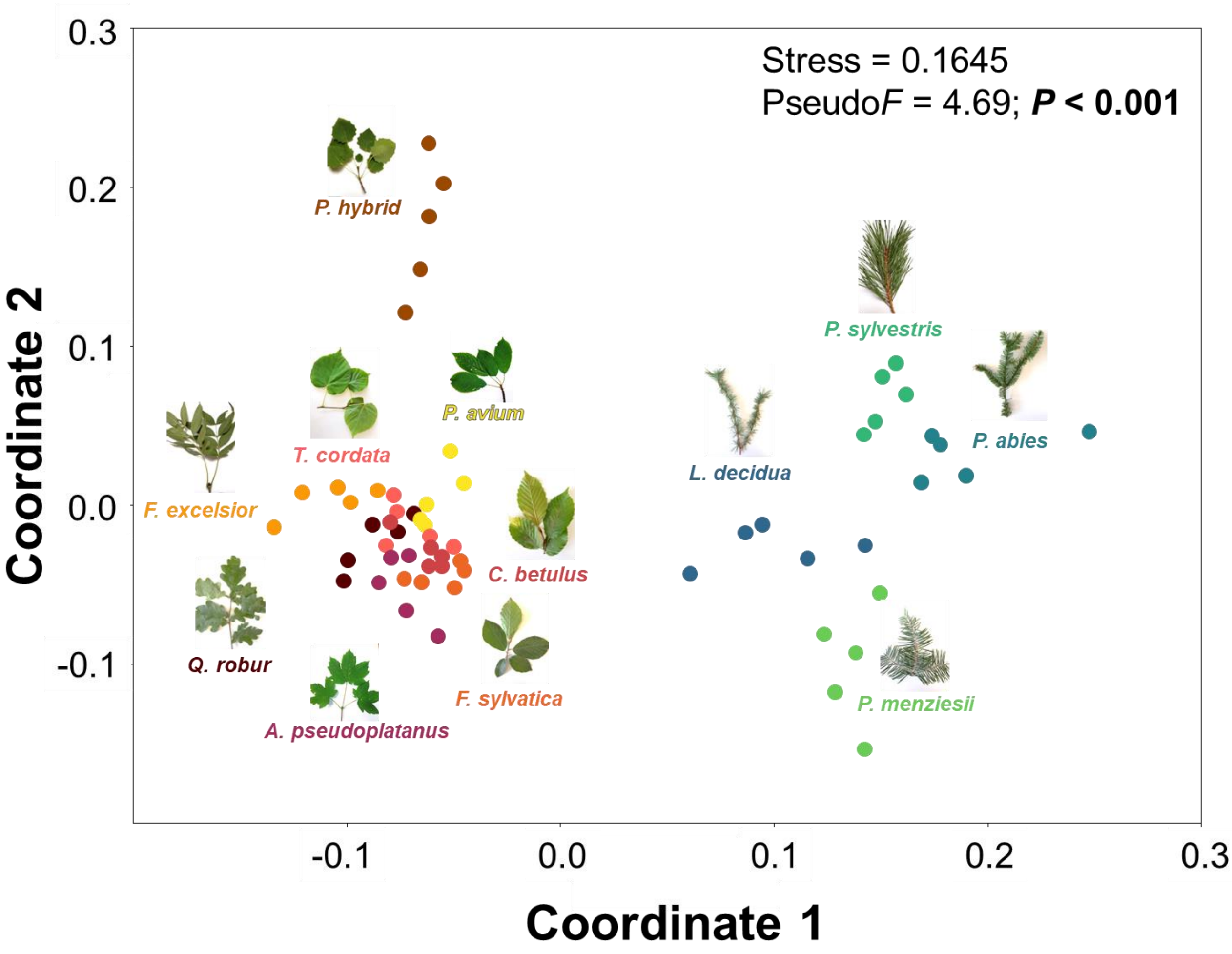

**Figure S2** Network between tree species and plant pathogenic fungal ASVs. Large circles indicate each tree species. Tree species name can be found at the outer layer of the network cycle. are Smaller circles in light purple color refer to plant pathogenic fungal ASVs.

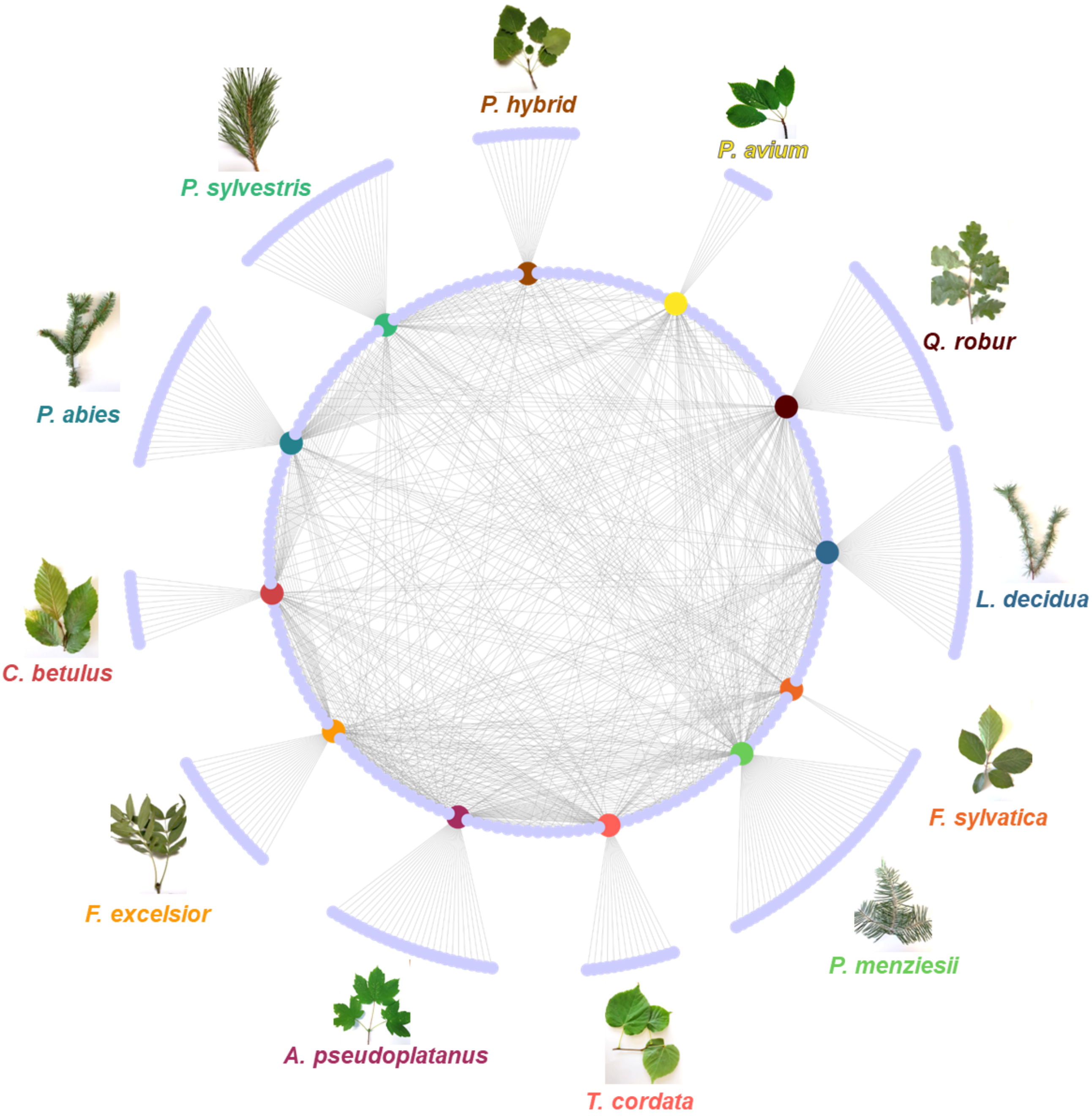

**Figure S3.** The taxonomic normalized stochasticity ratio (tNST) (A) and ecological stochasticity (B) in broadleaf and conifer trees. *, *P*<0.05

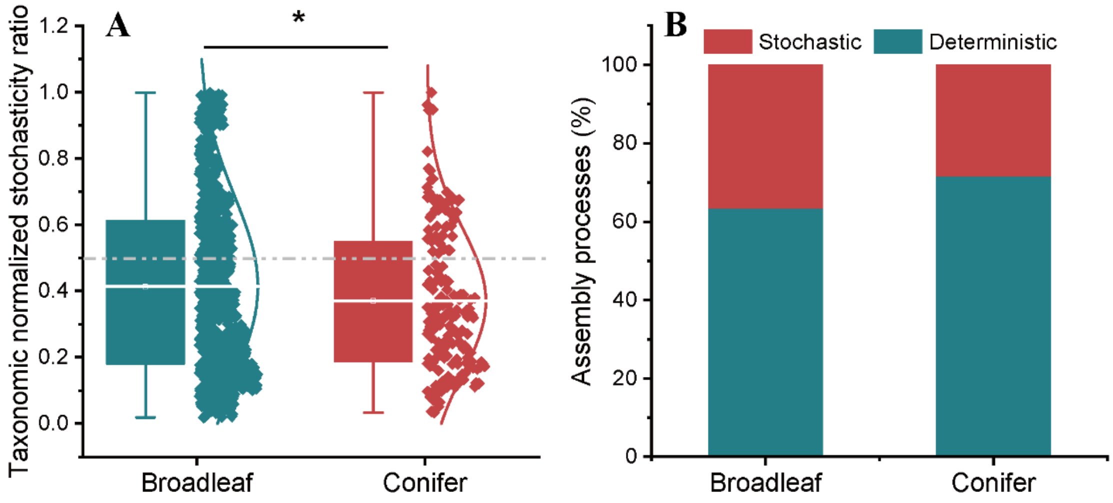

**Figure S4** Goodness-of-fit statistics ($R^2$) of environmental variables fitted to NMDS ordination of fungal plant pathogenic community based on presence/absence data and Jaccard distance measure (a), Venn diagrams showing the contributions of the factors shaping fungal plant pathogenic community (b–c). The locations (latitude and longitude) of each tree replicate are provided in Supplementary Table S4. Nutrient evaluated in the analysis of broadleaf trees was $NH_4^+$–N. Nutrients evaluated in the analysis of coniferous trees were DOC, $N_{org}$, K, Mg, and P. The number and percentage in the parentheses in the Venn diagram indicate the explained variance and its percentage in the total explainable variance.

**a**

| Presence/absence | Broadleaf | | Conifer | |
|---|---|---|---|---|
| | $R^2$ | $P$ | $R^2$ | $P$ |
| Tree species | **0.83** | **0.001** | **0.91** | **0.001** |
| Water content | 0.01 | 0.776 | 0.45 | **0.008** |
| pH | 0.45 | **0.001** | 0.60 | **0.002** |
| DOC | 0.11 | 0.123 | **0.71** | **0.001** |
| $NH_4^+$ – N | 0.35 | **0.001** | 0.07 | 0.548 |
| $NO_2^-$ – N | 0.03 | 0.570 | 0.26 | 0.051 |
| $NO_3^-$ – N | 0.00 | 0.997 | ND | ND |
| $N_{Min}$ | 0.12 | 0.070 | 0.07 | 0.530 |
| $N_{Org}$ | 0.10 | 0.139 | 0.41 | **0.004** |
| Ca | 0.12 | 0.109 | 0.20 | 0.146 |
| Fe | 0.09 | 0.154 | 0.10 | 0.414 |
| K | 0.12 | 0.115 | 0.38 | **0.015** |
| Mg | 0.04 | 0.480 | 0.51 | **0.001** |
| P | 0.06 | 0.303 | 0.37 | **0.024** |
| Latitude | 0.08 | 0.223 | **0.82** | **0.001** |
| Longitude | 0.05 | 0.325 | **0.91** | **0.001** |

*Bold letter indicates statistical significances
**ND = Not detected

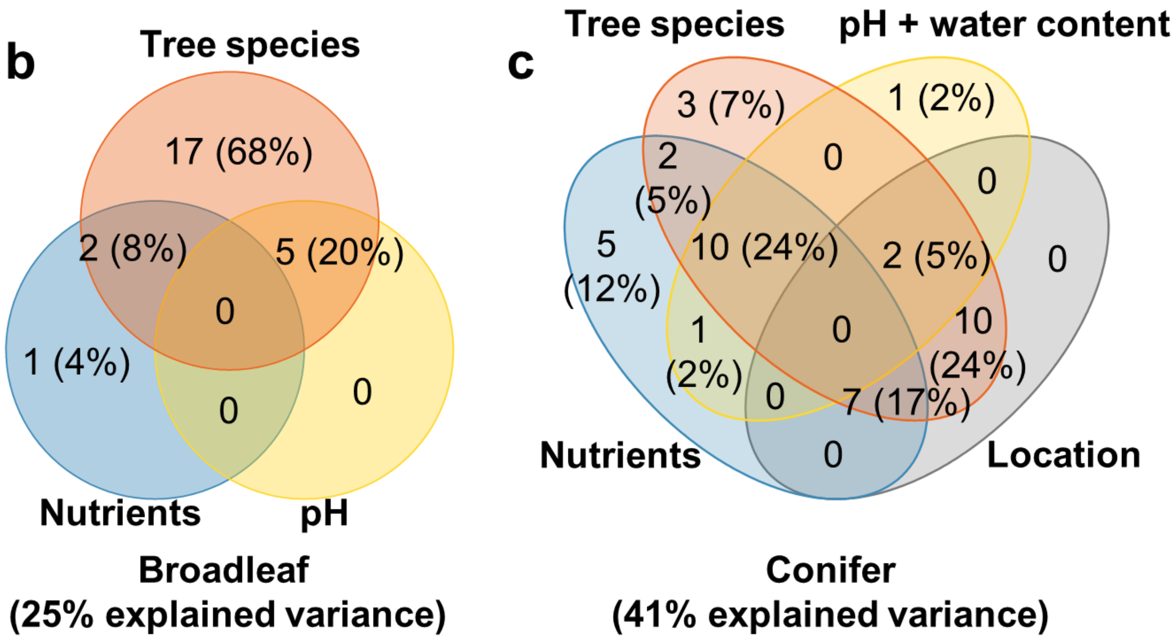

Supplement: Supplementary file 1 [file DataSheet_1.pdf]
